# Spectrum-Effect Relationships Between the Bioactive Ingredient of *Syringa oblata* Lindl. Leaves and Its Role in Inhibiting the Biofilm Formation of *Streptococcus suis*

**DOI:** 10.3389/fphar.2018.00570

**Published:** 2018-06-05

**Authors:** Yan-Yan Liu, Xing-Ru Chen, Ling-Fei Gao, Mo Chen, Wen-Qiang Cui, Wen-Ya Ding, Xue-Ying Chen, Bello-Onaghise God’spower, Yan-Hua Li

**Affiliations:** ^1^College of Veterinary Medicine, Northeast Agricultural University, Harbin, China; ^2^Heilongjiang Key Laboratory for Animal Disease Control and Pharmaceutical Development, Harbin, China

**Keywords:** *Syringa oblata* Lindl., spectrum-effect relationships, active ingredients, biofilm formation, computational studies

## Abstract

*Syringa oblata* Lindl. (*S. oblata*) has been used in herbal medicines for treating bacterial diseases. It is also thought to inhibit *Streptococcus suis* (*S. suis*) biofilm formation. However, due to the inherent nature of the complexity in its chemical properties, it is difficult to understand the possible bioactive ingredients of *S. oblata.* The spectrum-effect relationships method was applied to screen the main active ingredients in *S. oblata* obtained from Heilongjiang Province based on gray relational analysis. The results revealed that Sub-MICs obtained from 10 batches of *S. oblata* could inhibit biofilm formation by *S. suis*. Gray relational analysis revealed variations in the contents of 15 main peaks and rutin was discovered to be the main active ingredient. Then, the function of rutin was further verified by inhibiting *S. suis* biofilm formation using crystal violet staining. Computational studies revealed that rutin may target the chloramphenicol acetyltransferase protein in the biofilm formation of *S. suis.* In conclusion, this study revealed that the spectrum-effect relationships and computational studies are useful tools to associate the active ingredient with the potential anti-biofilm effects of *S. oblata*. Here, our findings would provide foundation for the further understanding of the mechanism of *S. oblata* intervention in biofilm formation.

## Introduction

To date, herbal medicines are gaining more and more attention in recent years because of the availability of multiple chemical ingredients (Yi et al., 2014). Among others, chemical fingerprint analysis has the potential of characterizing the key components and unknown components in a complex system (Lee et al., 2009). Nevertheless, it is very difficult to determine the main ingredients that provide the therapeutic effects in herbal medicines (Kong et al., 2011). It is well-known that spectrum-effect relationships analysis (Kong et al., 2009b) and chemical fingerprints have been used to determine the main ingredients in herbal medicines. In addition, spectrum-effect relationships which applied to the screening and analysis of active ingredients were characterized by similarity analysis, clustering analysis, principal component analysis (PCA), gray relational analysis, partial least squares, and so on (Shi et al., 2016). Among them, gray relational analysis has been certified to be a useful tool to deal with poor, incomplete, and uncertain information (Kuo et al., 2008). The gray system theory can be used to solve the complicated interrelationships among the multiple performance characteristics effectively (Lin and Lin, 2002). Thus, similarity analysis, clustering analysis, PCA and gray relational analysis may be able to elucidate the association between therapeutic effect and main component in herbal medicines.

To find the main ingredients, we chose Syringae Folium leaves as samples. Syringae Folium leaves are mainly from the dry leaves of Oleaceae plant: *Syringa oblata* Lindl. (*S. oblata*), *Syringa diatata* Nakai, and *Syringa vulgaris* L. Among them, *S. oblata* is widely distributed in Heilongjiang Province and has been proved to have a huge potential in the development of Chinese medicine through its plant resources, chemical constituents, pharmacological action, and clinical application (Bai et al., 2017). It is well-distributed in the temperate and frigid temperate regions of the middle and high latitudes of Eurasia and North America (Liu et al., 2010). There are also more than 20 species in China, beginning from the northeast to the southwest provinces (Su et al., 2015). Among the *Syringae* spp. found in the temperate regions of Heilongjiang Province, *S. oblata* is widely cultivated. It has strong vitality and complex chemical composition. It contains syringopicroside (Liu et al., 2010) and oleuropein (Bi et al., 2011). There are also other constituents such as flavonoids, e.g., rutin (Wang S. et al., 2017), as well as volatile oil compounds (Venturini et al., 2014).

In addition, the reports have shown that *S. oblata* has significant pharmacological properties such as anti-bacterial (Bai et al., 2017) and anti-inflammatory (Liu and Wang, 2011). It has also been reported that sub-MICs of aqueous extracts of *S. oblata* Lindl. decreases biofilm formation by *Streptococcus suis* (Bai et al., 2017). Using gray relational analysis, we could identify the main ingredients. Furthermore, Bai et al. (2017) have predicted that the up-regulated chloramphenicol acetyltransferase (CAT) protein of *S. suis* was possibly associated with biofilm formation (McLennan et al., 2008; Sahni et al., 2009). However, the detailed molecular interaction between the main ingredient and the 3D CAT protein is still unknown. Currently, computer docking analysis is an internationally recognized method employed in interpreting the relationship between bioactive ingredients and their perceived therapeutic effects (Mordalski et al., 2015; Haddad et al., 2016). Molecular docking is performed to predict the interaction between compounds and protein (Tulahong, 2015). If the 3D structure of CAT protein is constructed, we may identify the interaction between compounds and CAT protein by docking studies. It would lay the foundation for understanding the molecular mechanism involved in the inhibition of biofilm formation in *S. suis* by the main ingredient.

**FIGURE 1:**
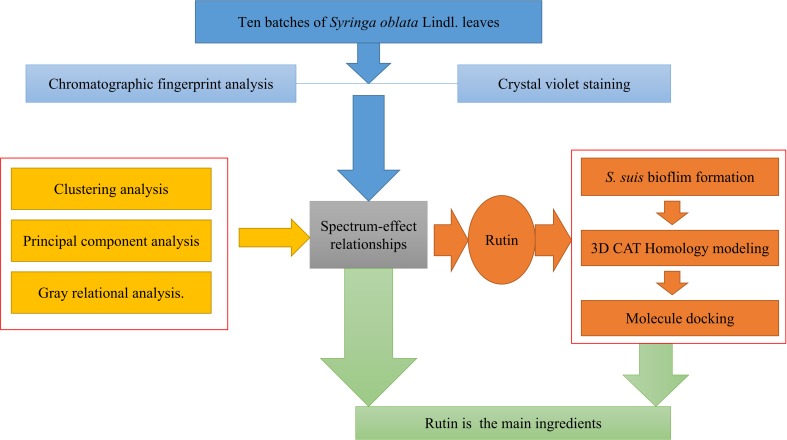
The experimental flow chart.

In this study, according to the established fingerprint, 10 batches of *S. oblata* collected from different regions (Heilongjiang Province, China) were analyzed and the main ingredients of *S. oblata* were screened by gray relational analysis. The main ingredient was verified by inhibiting the biofilm formation of *S. suis in* *vitro* and the docking-based virtual screening. Above all, the study offers a new approach for the determination of the active ingredients in *S. oblata* and the most effective substance against *S. suis* biofilm formation. The experimental flow chart of this study is shown in **Figure 1**.

## Materials and Methods

### Plant Materials

Ten batches of samples were collected from different regions in Heilongjiang Province of China in September, 2015 (**Table 1** and **Supplementary Material 1**), and their species were identified after drying the leaves at room temperature by Professor Mingxia Bai in Heilongjiang Academy of Agricultural Sciences Horticulture Branch. Then the samples were blended and sifted through the 24-mesh sieve.

**Table 1:** Representative samples of *S. oblata* in September, 2015 in Heilongjiang investigated in this study.

No.	Sample origin	Highest temperature (°C)	Lowest temperature (°C)	Average temperature (°C)
S1	Daqing	28	3	17
S2	Jiagedaqi	23	-3	12
S3	Harbin	26	4	16
S4	Hegang	28	0	15
S5	Jixi	27	2	15
S6	Jiamusi	27	1	16
S7	Qiqihaer	28	3	17
S8	Shuangyashan	26	1	15
S9	Mudanjiang	28	-1	16
S10	Zhaodong	28	2	17

### Chemical Analysis

Chromatographic fingerprint analysis was done using Waters Alliance HPLC system (Shimadzu, Corporation, Kyoto, Japan) that is equipped with a binary pump and a UV/V is detector. The chromatographic separation was performed on a Diamosil C18 column (4.6 mm × 250 mm, 5 μm) using aqueous solution of 0.1% formic acid (solvent A) and acetonitrile (solvent B) as mobile phase at a flow rate of 1 mL/min. The gradient program was 0 min, 5% solvent B; 30 min, 53% solvent B; and 35 min, 5% solvent B. The chromatogram was monitored at a wavelength of 245 nm during the experiment. The method was optimized by precision, repeatability, and stability of the target on the relative retention time and relative peak area in **Supplementary Material 2**.

All samples were completely dried at room temperature. Ten batches of samples of *S. oblata* leaves (leaves, 200 g) were boiled in deionized water (10 times of the amount of leaves) for 45 min, decanted, filtered and evaporated at 60°C. Aqueous extract of these dried *S. oblata* leaves was weighed and reconstituted to a concentration of 3 mg/mL. The samples were filtered through a 0.45 μm membrane filter prior to HPLC analysis (Bai et al., 2017).

Thereafter, all samples were analyzed by Similarity Evaluation System for Chromatographic Fingerprint of Traditional Chinese Medicine (Version 2004A) (Kong et al., 2009b) which is recommended by State Food and Drug Administration of China (SFDA). Clustering analysis and PCA are also very popular visualization tools in analyzing the samples and these were done by the software Metabo Analyst 3.0. Gray relational analysis which is a multi-factor statistical analysis method (Zhang and Zhang, 2007) was used to determine the correlation between the 15 common peaks of the fingerprint of each *S. oblata* leaf sample and the data obtained from the inhibition of *S. suis* biofilm. Using spectrum-effect analysis, fingerprinting, and biofilm formation on the 10 batches of *S. oblata* samples were analyzed. The experiment used “Gray Correlation Software (Version 3)” to calculate gray relational degree.

### Bacterial Strains and Culture Conditions

In this study, *S. suis* ATCC700794 strain was cultured in Todd Hewitt broth (THB) (THB: Summus, Ltd., Harbin, Heilongjiang, China) and Todd Hewitt broth agar (THA) supplemented with 5% (*v/v*) fetal bovine serum (Sijiqing, Ltd., Hangzhou, Zhejiang, China) at 37°C. The original THB was supplemented with 1.8% Bacto-agar (Difco Laboratories) in order to obtain THA. All the media were autoclaved for 30 min at 121°C. The strain was transferred from the stock cultures to THA prior to inoculation and incubated aerobically at 37°C for 16 h. Then, the strain was repeatedly subcultured under the same conditions. The final cultures were used for the minimum inhibitory concentration (MICs) assays and the biofilm formation assays.

### Determination of Minimum Inhibitory Concentration (MIC)

The MICs of the 10 batches of dried *S. oblata* samples and rutin standard solution of methanol (Guoyao, Ltd., China) were determined by Microdilution method (Yang et al., 2015). Overnight cultures of *S. suis* were diluted in sterile saline to a density of McFarland 0.5 standard which corresponds to 1 × 10^8^ colony-forming units (CFU)/mL. Thereafter, the cultures of *S. suis* were further diluted to 1:100 using sterile THB. Then 100 μL each of the samples and rutin were added to each well of a 96-well plate (Corning Costar^®^ 3599, Corning, NY, United States) in 100 μL culture medium, respectively. Control bacteria were cultivated in the absence of the 10 batches of dried *S. oblata* samples and rutin. After the incubation for 24 h at 37°C, the MICs were determined as the lowest concentration of the 10 batches of *S. oblata* samples and rutin.

### *S. suis* Biofilm Formation Assays

The *S. suis* biofilm formation was evaluated using crystal violet staining (Corning Costar^®^ 3599, Corning, NY, United States) (Yang et al., 2015). *S. suis* was grown in a Costar R 3599 96-well plate in the presence of the 10 batches of 1/2 MIC of *S. oblata* samples and 1/2 MIC, 1/4 MIC, and 1/8 MIC of rutin. Negative and positive control wells contained culture medium and bacterial suspension, respectively. Biofilms were treated as described previously (Yang et al., 2015). The plates were incubated for 24 h at 37°C. Then, the remaining bacteria were aspirated and washed three times with 0.25 mL of sterile physiological saline. The remaining attached bacteria were fixed with 0.2 mL of 99% methanol (Guoyao, Ltd., China) per well in 15 min and 0.2 mL of 2% crystal violet (Guoyao, Ltd., China). After the plates were air dried, the dye was resolubilized with 0.2 mL of 33% (*v/v*) glacial acetic acid (Guoyao, Ltd., China) per well. Using a microtiter plate reader (DG5033A, Huadong, Ltd., Nanjing, Jiangsu, China), the OD of each well was measured at 570 nm.

### Computational Studies

In order to determine the main ingredients of *S. oblata* samples targeting the formation of *S. suis* biofilm, we constructed the 3D structure of CAT. Although there was no available crystal structure of the CAT protein from *S. suis*, the amino acid sequence of CAT was retrieved from UniProt (Accession No. C6GT52). It is important to construct the 3D structure based on the known structures by homology modeling method as described previously (Chen et al., 2017). Then, a series of homology methods including sequence similarity searches, target sequences were constructed. The total energy of lowest probability density functions (PDFs) and discrete optimized potential energy (DOPE) score were performed in order to construct an appropriate 3D model of CAT protein of *S. suis* using Discovery Studio (version 3.0). Thereafter, the best model was assessed and verified. Finally, molecular docking calculations were performed with DS-LibDock protocol under default conditions (Chen et al., 2017).

### Statistical Analysis

All the experiments were performed in triplicates. Data analysis and calculation of standard deviation were done using SPSS 11.0.0 (IBM, United States).

## Results

### HPLC Fingerprints of *S. oblata* Samples

**FIGURE 2:**
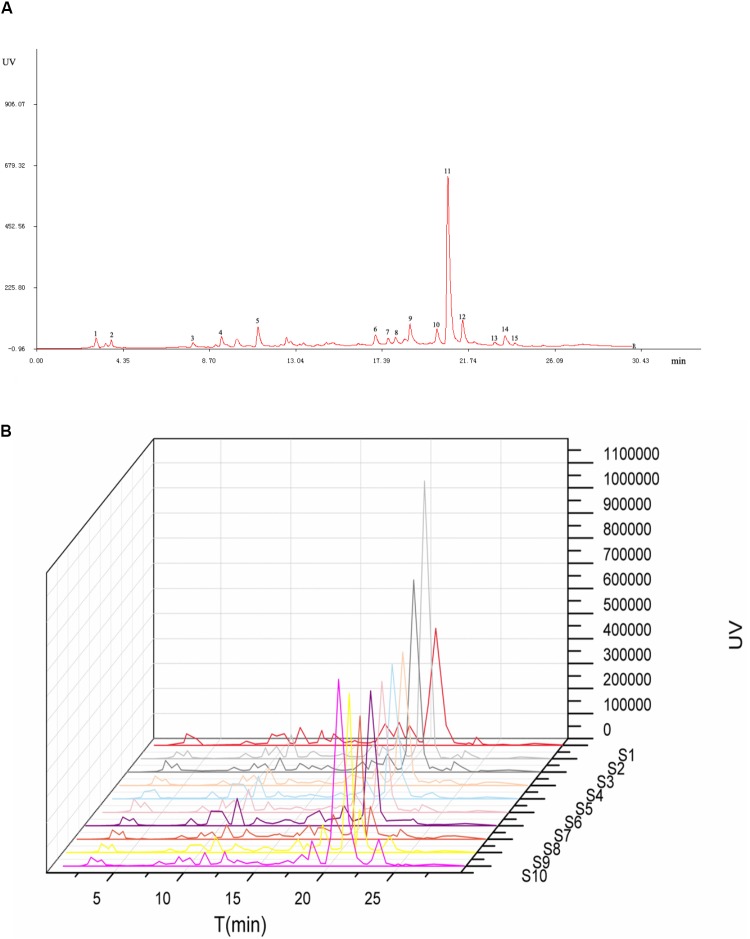
Chromatographic fingerprints for reference **(A)** and all the *Syringa oblata* sample **(B)**.

**FIGURE 3:**
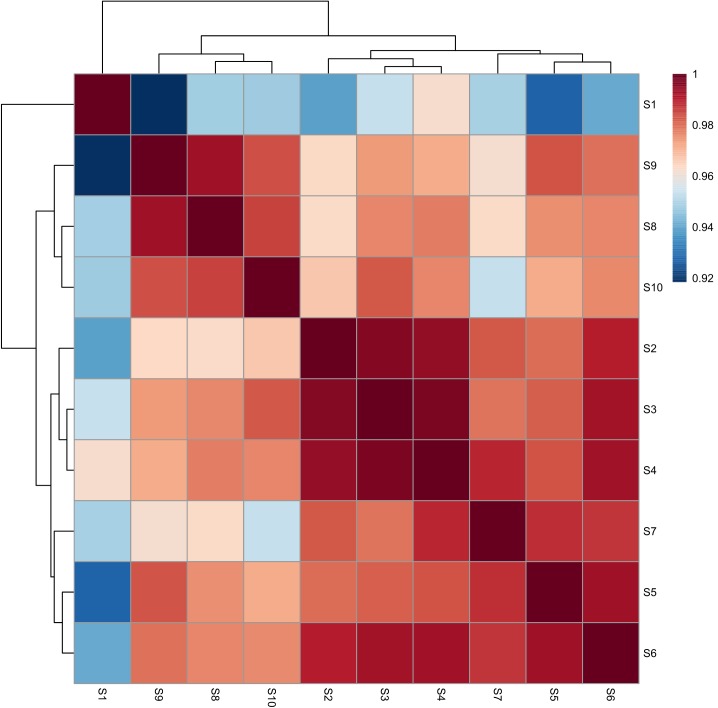
The correlation coefficient for all the *S. oblata* sample.

The HPLC fingerprints of *S. oblata* samples are shown in **Figure 2**. The results revealed that there were differences in the fingerprints among the 10 batches of *S. oblata* samples (**Figure 2B**). Thereafter, the professional similarities were calculated using the Similarity Evaluation System for Chromatographic Fingerprint of herbal medicines (Version 2004A) (Kong et al., 2009b). Similarities between the reference fingerprint (**Figure 2A**) and each chromatographic profile of the 10 batches of *S. oblata* samples (**Figure 2B**) were evaluated by calculating the correlation coefficient in **Figure 3**. These results indicated that S1 and S2 which were located in oil field with poor soil conditions and a low temperature region respectively had the lowest degree of similarity (Liao et al., 2015). This may be responsible for the differences in the fingerprints of *S. oblata.* Despite this, these results revealed that the 10 batches of samples of *S. oblata* from different locations shared nearly the same correlation coefficients (over 0.92) of similarities and similar chemical constituents.

### Clustering Analysis

**FIGURE 4:**
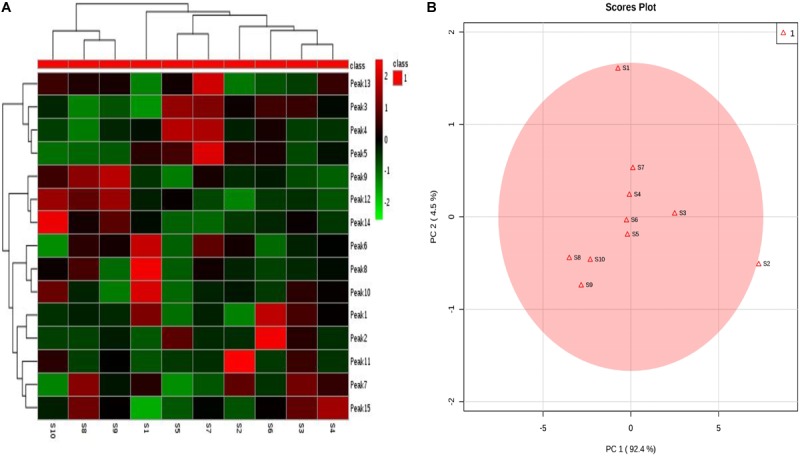
*Syringa oblata* samples from different sources: **(A)** heatmaps of 15 constituent content; **(B)** principal component analysis (PCA) results.

Clustering analysis is one of the detection and quantification classification evaluation methods that is commonly used in analyzing samples of herbal medicines (Kong et al., 2009a). Clustering analysis method is well-known and has gained wide range application in fingerprint analysis. This is because its application is simple and its method of data interpretation is non-parametric (Zou et al., 2005). The 10 samples were analyzed using clustering analysis (Dong et al., 2017) of the peak areas of the 15 common analytical markers in the HPLC fingerprints. Results of cluster analysis showed that the fingerprints separated into seven clusters, as shown in **Figure 4A**. Cluster 1 contained only S10; cluster 2 included S8 and S9; cluster 3 included only S1; cluster 4 included S5 and S7; cluster 5 contained only S2; cluster 6 contained only S6 and cluster 7 contained S3 and S4. These results could be explained as follows: the differences in the chemical ingredients could be related to the different geographic locations. Interestingly, the results of clustering analysis were consistent with those of similarity analysis.

### Principal Component Analysis

In addition, the results of PCA are presented in **Figure 4B**. The first two principal components (PC1 and PC2) revealed the clear differences in the cultivating locations, which accounted for the 92.4% and 4.5% of the total variability. From **Figure 4B**, it could be seen that the samples were clearly divided into five groups: group 1 (S4, S5, S6, S7); group 2 (S8, S9, S10); group 3 (S1); group 4 (S2); and group 5 (S3). PCA could further be used to evaluate the information of the fingerprints and provide reference to assess the quality of different batches of *S. oblata*. The differences in the results revealed that the uniqueness in the compositions of the samples of *S. oblata* may be due to the different environmental conditions in which the plants were exposed. In particular, it was clear from the map (**Supplementary Material 1**) that groups 3, 4, and 5 were far apart from groups 1 and 2. Interestingly, S2 came from a low temperature region, S3 was obtained from the capital city of Heilongjiang (with a high-rise building region in the southwest) and S1 was obtained from the south western region (oil field) with poor soil conditions with little difference from other regions, proving the feasibility of PCA. Furthermore, PCA had helped in effectively guiding the study and understanding of the active ingredients in *S. oblata*. In spite of this, no difference was observed in the results of clustering analysis and similarity analysis among the 10 batches of *S. oblata*.

### Spectrum-Effect Relationship

**FIGURE 5:**
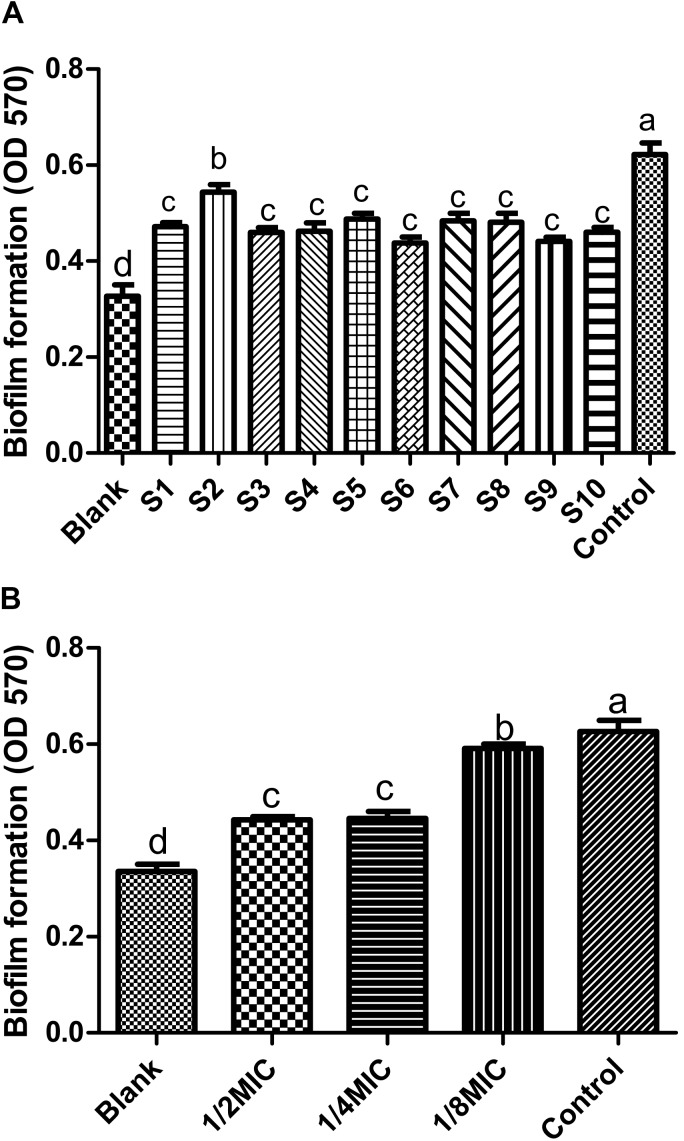
Inhibitory effect on biofilm formation by *Streptococcus suis* (*n* = 5, x ± SD): **(A)** 10 batches of *S. oblata* samples; **(B)** the rutin standard. Different letters indicate a significant difference at *p* < 0.05.

In order to find out the main active ingredients involved in the inhibition of biofilm formation, 10 batches of *S. oblata* samples were evaluated in *S. suis* biofilm. The MICs were determined as 12.5 mg/mL, except for S2 and S9 which were determined as 6.25 mg/mL. The results revealed that the 1/2 MIC of 10 batches of the *S. oblate* samples had the ability to significantly inhibit the formation of *S. suis* biofilm (*p* < 0.05) (**Figure 5A**) compared with positive control. Among them, S2, which was obtained from a low temperature region, was different from other regions in inhibiting the formation of *S. suis* biofilm. In addition, gray relational analysis was used to determine the correlation between the 15 common peaks of fingerprint and *S. suis* biofilm data of each *S. oblata* sample. The results revealed that the characteristic peak 6 was the greatest gray relational rankings in **Table 2**. It was closely related to *S. suis* biofilm formation and might be the main pharmaceutical component of *S. oblata* which inhibited the *S. suis* biofilm. Previous studies have shown that the main component in characteristic peak 6 was rutin at the retention time of 17.66 min by HPLC-ESI-MS analysis (Bai et al., 2017). Thus, the rutin was evaluated to monitor its effect against biofilm formation by *S. suis*. The results revealed that the MIC of rutin against *S. suis* was 0.3125 mg/mL. The inhibitory effect of rutin was significant for 1/2 MIC and 1/4 MIC (*p* < 0.05), with no effect by 1/8 MIC compared with positive control (**Figure 5B**). The contents of rutin in 10 batches of *S. oblata* in MICs are shown in **Supplementary Material 2**.

**Table 2:** Gray relational grade of the peak with biofilm formation.

No.	Gray relation	Gray relational rankings
1	0.777193	12
2	0.709735	13
3	0.881281	4
4	0.901884	2
5	0.900460	3
6	0.905249	1
7	0.864141	6
8	0.876266	5
9	0.859622	7
10	0.799613	11
11	0.841602	9
12	0.682051	14
13	0.845761	8
14	0.521954	15
15	0.896398	10

### Computational Studies

**FIGURE 6:**
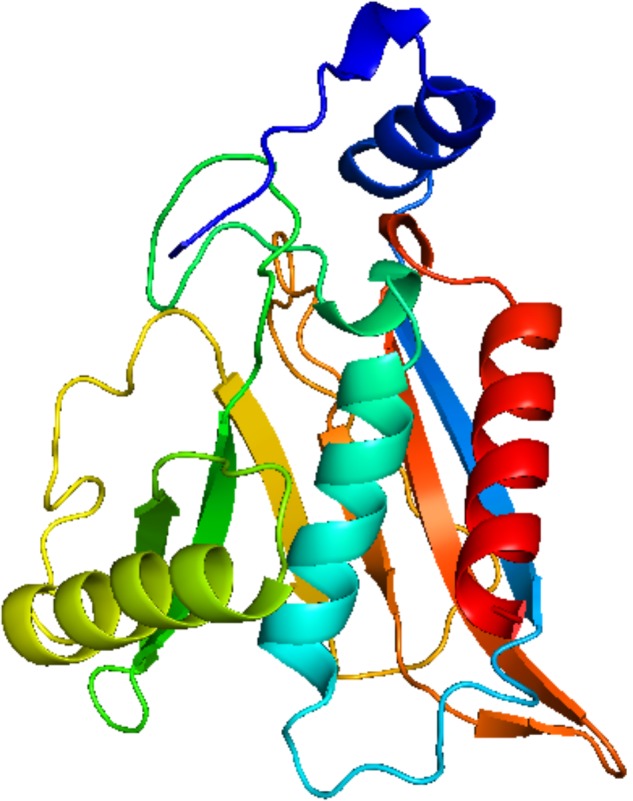
Modeled structure of CAT protein from *Streptococcus suis.*

**FIGURE 7:**
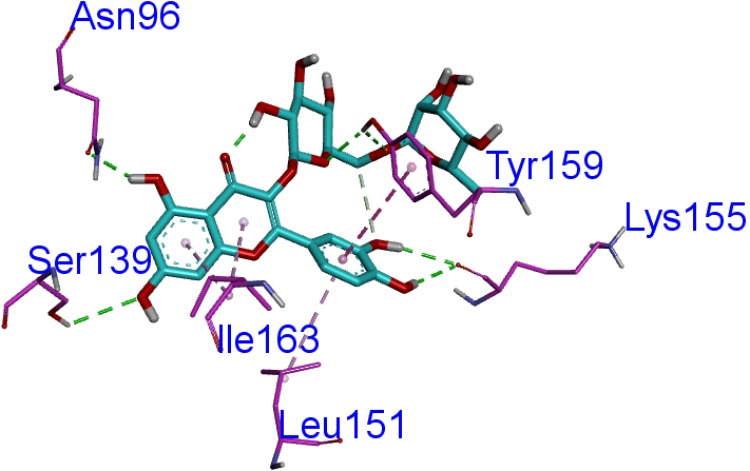
The ligand of rutin compared by the LibDock docking method. The atoms of the compound, rutin, are shown in ball-and-stick format and colored by atom. Polar interactions are shown as dashed colored lines. Among them, the green dotted line represents the hydrogen bond interaction. The pink dotted line represents *p-π* interactions interaction.

Homology modeling of CAT protein was conducted. The 3D structure of CAT from *Escherichia coli* (PDB code: 3CLAA), *Escherichia coli* (PDB code: 1Q23L) and *Bacteroides thetaiotaomicron* (PDB code: 2I9DC) were screened as the template structure. The best homology model was established based on the least PDF total energy (11550.45) and DOPE score (-23049.50). The final model which had the maximum number of residues in the favored regions (93.26%) was selected. Indeed, the Z-score calculated for the template was -1.56. The results demonstrated that the established homology model of the CAT should be a reasonable structure. The results are presented in **Supplementary Material 3**. Thereafter, the active-site was copied from *Escherichia coli* (PDB code: 3CLAA). Finally, the results revealed that rutin could interact with CAT protein having a LibDock score (100.31) by docking analysis. From the results, we observed that rutin targets the CAT protein with three hydrogen bonds and two *p-π* interactions (**Figures 6**, **7**). Above all, we observed that rutin could combine with CAT protein to inhibit biofilm formation by *S. suis*.

## Discussion

Herbal medicines have been widely used for treating bacterial diseases (Wong et al., 2010). It has a long therapeutic history over thousands of years (Chang et al., 2008; Kong et al., 2009a). *S. suis* is one of the leading sources of high economic losses in the pig industry worldwide, which causes a wide variety of disease, including meningitis, arthritis, septicemia and bacterial diseases (Wang et al., 2011, 2016). In addition, it is important to control the formation of *S. suis* biofilm in the fight against bacterial diseases in pigs (Wang et al., 2012). Recent studies have shown that *Leptospermum scoparium* (manuka) (Song et al., 2013), *Glycyrrhiza* (Guldas et al., 2016), and *Punica granatum* L. plants (Bakkiyaraj et al., 2013) can also inhibit biofilm formation. Furthermore, it has also been reported that aqueous extract of sub-MICs of *S. oblata* Lindl. aqueous extract decreased biofilm formation in *S. suis* (Bai et al., 2017). In this study, a crystal violet staining (Yang et al., 2015) was used to evaluate the anti-biofilm effects of *S. suis*. The results indicated that 1/2 MIC derived from 10 batches of dried *S. oblata* samples significantly decreased the biofilm formation capability of *S. suis.* This is in consonance with previous study (Bai et al., 2017). However, this plant material is made up of complex chemical composition. Using chemical fingerprints, it was difficult to determine active ingredient in *S. oblata* that was responsible for inhibiting biofilm formation among the chemical constituents (Wang Y. et al., 2017).

Thus, we used spectrum-effect relationships analysis (Kong et al., 2009b) to characterize the bioactive substance in *S. oblata* samples that inhibited *S. suis* biofilm formation. First, the results revealed that the combination of similarity analysis, clustering analysis, and PCA was able to effectively classify the samples objectively in accordance with different factors, such as geographical locations, elevation and the climatic conditions they were exposed to. Although the chemical fingerprints could show the differences between most of the constituents, it could not directly show the anti-biofilm effect of *S. oblata.* Thus, the gray relational analysis combined with the study of the effect of the chemical components on biofilm formation was used to obtain the bioactive ingredients. At the same time, gray relational analysis could explore the correlation of fingerprints and the anti-inflammatory effects directly (Lu et al., 2017) and be used to evaluate the multiple performance characteristics of the bioactive substance and then be optimized into a single gray relational grade (Lin and Lin, 2002). Thus, it was analyzed with *S. suis* biofilm data of each *S. oblata* sample and the peak area of 15 constituents. In our study, the characteristic peak 6 played an important role in the inhibition of *S. suis* biofilm formation. This proved that rutin, which corresponded to the characteristic peak 6, was the potential substance that inhibited the formation of *S. suis* biofilm, and this finding is in agreement with earlier findings previously reported by Bai et al. (2017). There is a direct relationship between the content of rutin and its ability to inhibit *S. suis* biofilm. Kong et al. (2017) also reported that the most abundant compounds might not be the major contributors to antibacterial activity. This is also in tandem with our study. However, it was unclear how rutin inhibited *S. suis* biofilm formation.

Previous study has reported that some differentially expressed proteins may be involved with the formation and inhibition of biofilm by *S. suis* for relative and absolute quantitation (iTRAQ) labeling (Bai et al., 2017). It was discovered that the up-regulated CAT protein may inhibit the biofilm information mechanism of bacteria (McLennan et al., 2008; Sahni et al., 2009). The decreased levels of acetyl coenzyme A (acetyl-CoA) (Potrykus and Wegrzyn, 2001) was shown in CAT-expressing cells and acetyl-CoA played a role in biofilm formation (Pruess et al., 2010). Thus, in order to discover the active ingredients of *S. oblata* responsible for the mechanism involved in the inhibition of biofilm formation by *S. suis*, docking calculations were performed with DS-LibDock protocol using DS 3.0, which revealed that rutin could interact well with CAT protein which was built by homology modeling. Then the active-site was copied from *Escherichia coli* (PDB code: 3CLAA). Finally, the results from docking analysis proved that rutin could interact with CAT protein with LibDock score (100.31). The rutin attracted the CAT protein in *S. suis* through specific residual interaction with three hydrogen bonds of ASN 96, TYR 159, and SER 139 of CAT. In addition, there were also two *p-π* interactions of ILE 163 and LEU 151 with CAT acid side-chains (**Figures 6**, **7**). It may be possible to enhance CAT thermostability via a rational design approach and optimize the amino acid configuration surrounding (Kim et al., 1997). Our results speculate that rutin can also enhance the stability of the CAT and inhibit *S. suis* biofilm. Interestingly, this is the first report that rutin targets the CAT protein in *S. suis* biofilm.

## Conclusion

Ten batches of *S. oblata* from Heilongjiang province were analyzed using chemical fingerprint analysis. Then, spectrum-effect relationships confirmed rutin as the main bioactive ingredient in *S. oblate* which inhibits biofilm formation by *S. suis*. Molecular docking revealed that rutin targeted the CAT protein in *S. suis* biofilm thus leading to its inhibition. The novelty of this work is the identification of rutin as the active ingredient of *S. oblata* by spectrum-effect relationships and computational studies. Our findings here would provide the foundation for the further understanding of the mechanism of *S. oblata* intervention in biofilm formation.

## Author Contributions

Y-HL designed the whole experiment. Y-YL and X-RC directed the completion of the experiment. L-FG, MC, W-QC, W-YD, X-YC, and B-OG were supportive during the experiment.

## Conflict of Interest Statement

The authors declare that the research was conducted in the absence of any commercial or financial relationships that could be construed as a potential conflict of interest.
