# Supplementary material for: Spectrum-Effect Relationships Between the Bioactive Ingredient of Syringa oblata Lindl. Leaves and Its Role in Inhibiting the Biofilm Formation of Streptococcus suis
Source: Front Pharmacol. 2018 Jun 5;9:570. doi: 10.3389/fphar.2018.00570 (PMC5996274; doi:10.3389/fphar.2018.00570)

Supplementary Material 1

**Spectrum-effect Relationships between the Bioactive Ingredient of *Syringa oblata* Lindl. Leaves and its Role in Inhibiting the Biofilm Formation of *Streptococcus suis***

*Yan-Yan Liu**^1, 2+^, Xing-Ru Chen^1, 2+^, Ling-Fei Gao^1, 2^, Mo Chen^1, 2^, Wen-Qiang Cui^1, 2^, Wen-Ya Ding^1, 2^, Xue-Ying Chen^1, 2^, Bello-Onaghise God'spower^1, 2^, Yan-Hua Li^1, 2*^*

*^1College of Veterinary Medicine, Northeast Agricultural University, Harbin, Heilongjiang 150030^*

*^2Heilongjiang Key Laboratory for Animal Disease Control and Pharmaceutical Development, Harbin, China^*

*Correspondence to: Professor Yanhua Li, College of Veterinary Medicine, Northeast Agricultural University, 600 Changjiang Road, Xiangfang, Harbin, Heilongjiang 150030, P.R. China

Tel：+86 451 55191881

E‑mail: liyanhua1970@163.com (Y.-H.Li).

^†^These authors have contributed equally to this study and share first authorship.

**Supplementary Figure 1.** Location of *S. oblata* samples collected from different regions of Heilongjiang Province.


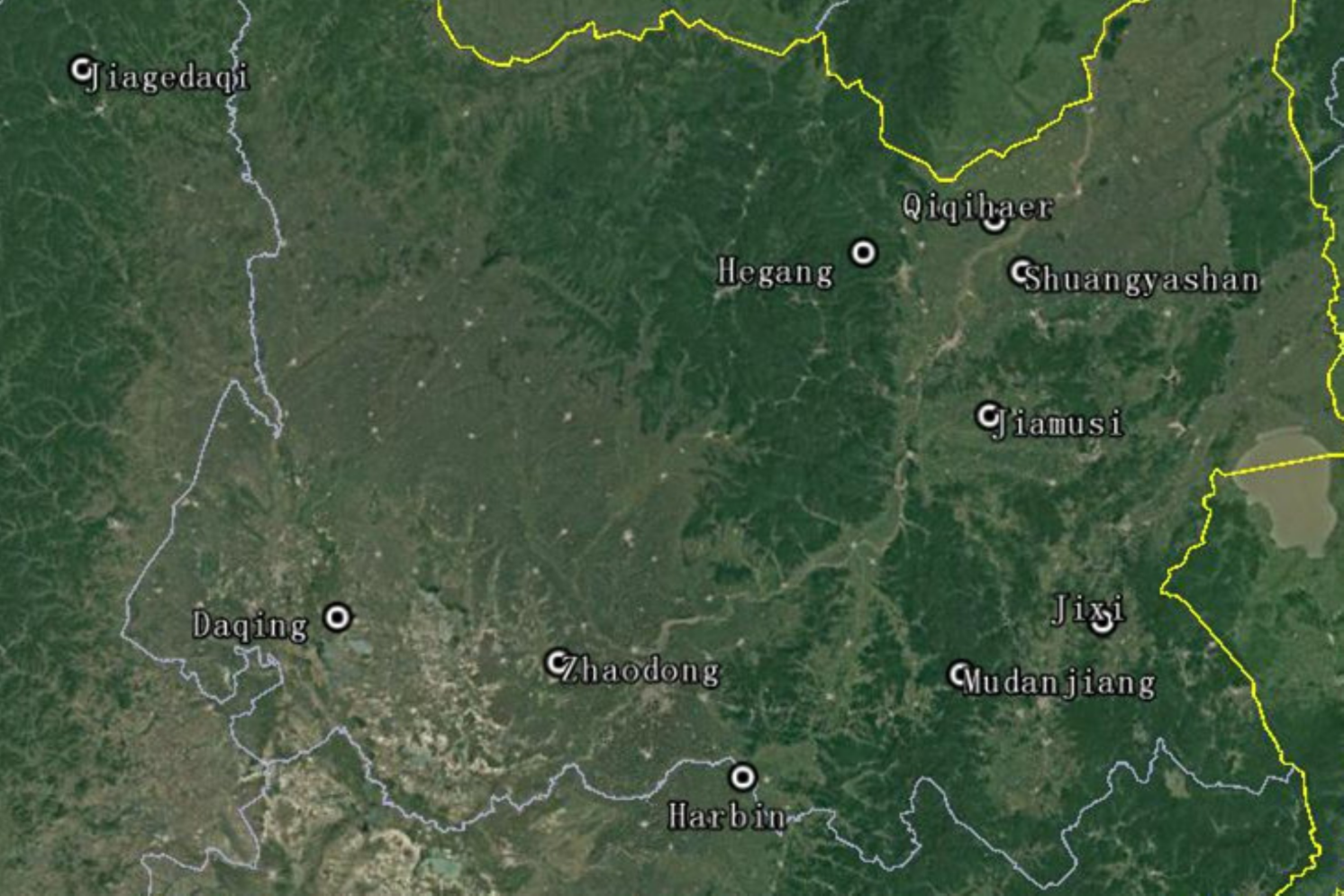

Supplement: Supplementary file 1 [file Data_Sheet_1.docx]
