# Supplementary material for: Spectrum-Effect Relationships Between the Bioactive Ingredient of Syringa oblata Lindl. Leaves and Its Role in Inhibiting the Biofilm Formation of Streptococcus suis
Source: Front Pharmacol. 2018 Jun 5;9:570. doi: 10.3389/fphar.2018.00570 (PMC5996274; doi:10.3389/fphar.2018.00570)
Supplement: Supplementary file 2 [file Data_Sheet_2.docx]

Supplementary Material 2

**Spectrum-effect Relationships between the Bioactive Ingredient of *Syringa oblata* Lindl. Leaves and its Role in Inhibiting the Biofilm Formation of *Streptococcus suis***

*Yan-Yan Liu**^1, 2+^, Xing-Ru Chen^1, 2+^, Ling-Fei Gao^1, 2^, Mo Chen^1, 2^, Wen-Qiang Cui^1, 2^, Wen-Ya Ding^1, 2^, Xue-Ying Chen^1, 2^, Bello-Onaghise God'spower^1, 2^, Yan-Hua Li^1, 2*^*

*^1College of Veterinary Medicine, Northeast Agricultural University, Harbin, Heilongjiang 150030^*

*^2Heilongjiang Key Laboratory for Animal Disease Control and Pharmaceutical Development, Harbin, China^*

*Correspondence to: Professor Yanhua Li, College of Veterinary Medicine, Northeast Agricultural University, 600 Changjiang Road, Xiangfang, Harbin, Heilongjiang 150030, P.R. China

Tel：+86 451 55191881

E‑mail: liyanhua1970@163.com (Y.-H.Li).

^†^These authors have contributed equally to this study and share first authorship.

**Supplementary Figure 1.** Chromatograms of *S. oblata* leaves samples on different columns: A: Spherisorb C18 column (4.6 mm x 250 mm, 5 μm); B: Diamosil C18 column (4.6 mm x 250 mm, 5 μm); C: Wondasil C18 column (4.6 mm x 150 mm, 5 μm)

A

B

C

**Supplementary Figure 2.** Chromatograms of *S. oblata* leaves samples on different mobile-phase systems: A: Methanol - H_2_O solution; B: Methanol - 1% HCOOH; C: Methanol -0.l% HCOOH solution; D: Acetonitrile - H_2_O solution E: Acetonitrile - 1% HCOOH solution; F: Methanol - 0.l% HCOOH solution.

A

B

C

D

E

F

**Supplementary** **Table 1.** Precision of the targets on the relative retention time (RRT). The relative standard deviations (RSD) of the average RRT were less than 1%.

| RRT | 1 | 2 | 3 | 4 | 5 | ‾x ± SD | RSD（%） |
| --- | --- | --- | --- | --- | --- | --- | --- |
| 1 | 0.16681 | 0.16548 | 0.16743 | 0.16631 | 0.16532 | 0.16676±0.00089 | 0.53 |
| 2 | 0.22158 | 0.22431 | 0.22443 | 0.22431 | 0.22449 | 0.22382±0.0013 | 0.56 |
| 3 | 0.44635 | 0.44531 | 0.44832 | 0.44356 | 0.44567 | 0.44584±0.0017 | 0.39 |
| 4 | 0.57474 | 0.57547 | 0.57534 | 0.57552 | 0.57507 | 0.57522±0.0003 | 0.06 |
| 5 | 0.66167 | 0.66125 | 0.66132 | 0.66153 | 0.66145 | 0.66144±0.0002 | 0.03 |
| 6 | 1.02749 | 1.02714 | 1.02725 | 1.02719 | 1.02736 | 1.02729±0.0001 | 0.01 |
| 7 | 1.05233 | 1.05195 | 1.04725 | 1.05195 | 1.05217 | 1.05113±0.0022 | 0.21 |
| 8 | 1.08636 | 1.08572 | 1.08587 | 1.08578 | 1.08611 | 1.08597±0.0003 | 0.02 |
| 9 | 1.16749 | 1.16669 | 1.16676 | 1.16663 | 1.16694 | 1.16690±0.0003 | 0.03 |
| 10 | 1.19164 | 1.19054 | 1.19061 | 1.19059 | 1.19101 | 1.19088±0.0005 | 0.04 |
| 11 | 1.23192 | 1.23065 | 1.23071 | 1.23071 | 1.23119 | 1.23104±0.0005 | 0.04 |
| 12 | 1.30998 | 1.30776 | 1.30652 | 1.30812 | 1.30561 | 1.30760±0.0017 | 0.13 |
| 13 | 1.33929 | 1.33886 | 1.33657 | 1.33597 | 1.33621 | 1.33738±0.0014 | 0.10 |
| 14 | 1.36760 | 1.36553 | 1.36721 | 1.36235 | 1.26872 | 1.36588±0.0014 | 0.10 |

**Supplementary Table 2.** Precision of the targets on the relative peak area (RPA). The relative standard deviations (RSD) of the average RPA were less than 3%.

| RPA | 1 | 2 | 3 | 4 | 5 | ‾x ± SD | RSD（%） |
| --- | --- | --- | --- | --- | --- | --- | --- |
| 1 | 0.14463 | 0.14389 | 0.14522 | 0.14517 | 0.15333 | 0.14645±0.0039 | 2.65 |
| 2 | 0.73716 | 0.73125 | 0.70603 | 0.75002 | 0.71526 | 0.72794±0.0175 | 2.40 |
| 3 | 0.38035 | 0.37093 | 0.39220 | 0.37362 | 0.38111 | 0.37964±0.0083 | 2.17 |
| 4 | 0.66304 | 0.63009 | 0.62956 | 0.63639 | 0.64399 | 0.64061±0.0138 | 2.16 |
| 5 | 0.29551 | 0.28099 | 0.29851 | 0.30366 | 0.30001 | 0.29573±0.0088 | 2.95 |
| 6 | 0.60244 | 0.57096 | 0.58649 | 0.60153 | 0.59958 | 0.59220±0.0135 | 2.28 |
| 7 | 1.00089 | 0.99314 | 0.96601 | 1.03402 | 1.03375 | 1.00556±0.0289 | 2.88 |
| 8 | 1.04673 | 0.99633 | 0.99916 | 1.02746 | 1.04202 | 1.02234±0.0236 | 2.31 |
| 9 | 0.72569 | 0.71582 | 0.69371 | 0.71711 | 0.71488 | 0.71344±0.0118 | 1.66 |
| 10 | 3.04102 | 3.11193 | 3.09839 | 3.16145 | 3.26566 | 3.13569±0.0844 | 2.69 |
| 11 | 0.46381 | 0.44913 | 0.43346 | 0.46159 | 0.46599 | 0.45479±0.0106 | 2.99 |
| 12 | 1.11521 | 1.13018 | 1.15953 | 1.11583 | 1.11583 | 1.12731±0.0190 | 1.69 |
| 13 | 1.15001 | 1.13968 | 1.15231 | 1.15962 | 1.14759 | 1.14984±0.0072 | 0.63 |
| 14 | 1.6226 | 1.14772 | 1.15935 | 1.15829 | 1.14307 | 0.15414±0.0082 | 0.72 |

**Supplementary Table 3.** Repeatability of the targets on the relative retention time (RRT). The relative standard deviations (RSD) of the average RRT were less than 1%.

| RRT | 1 | 2 | 3 | 4 | 5 | ‾x ± SD | RSD（%） |
| --- | --- | --- | --- | --- | --- | --- | --- |
| 1 | 0.16548 | 0.16436 | 0.16569 | 0.16445 | 0.16321 | 0.16464±0.0009 | 0.60 |
| 2 | 0.22510 | 0.22454 | 0.22523 | 0.22570 | 0.22583 | 0.22530±0.0005 | 0.21 |
| 3 | 0.4473 | 0.44259 | 0.44567 | 0.44623 | 0.44682 | 0.44572±0.0019 | 0.42 |
| 4 | 0.57469 | 0.57476 | 0.57507 | 0.53741 | 0.57474 | 0.56733±0.0167 | 0.95 |
| 5 | 0.66092 | 0.66089 | 0.66128 | 0.66586 | 0.66106 | 0.66200±0.0022 | 0.33 |
| 6 | 1.02729 | 1.02735 | 1.02728 | 1.02727 | 1.02690 | 1.02722±0.0002 | 0.02 |
| 7 | 1.05197 | 1.05215 | 1.05207 | 1.05195 | 1.05217 | 1.05192±0.0002 | 0.02 |
| 8 | 1.08583 | 1.08614 | 1.08604 | 1.05159 | 1.08564 | 1.08592±0.0002 | 0.02 |
| 9 | 1.16673 | 1.16705 | 1.16687 | 1.16695 | 1.16647 | 1.16681±0.0002 | 0.02 |
| 10 | 1.19073 | 1.1910 | 1.19091 | 1.19121 | 1.19031 | 1.19084±0.0003 | 0.03 |
| 11 | 1.23084 | 1.23123 | 1.23101 | 1.23141 | 1.23036 | 1.23097±0.0004 | 0.33 |
| 12 | 1.30887 | 1.30566 | 1.30677 | 1.30333 | 1.30757 | 1.30644±0.0019 | 0.14 |
| 13 | 1.33852 | 1.33563 | 1.33768 | 1.33676 | 1.33356 | 1.33643±0.0017 | 0.13 |
| 14 | 1.35360 | 1.35668 | 1.35785 | 1.35821 | 1.35623 | 1.35651±0.0016 | 0.12 |

**Supplementary Table 4.** Repeatability of the targets on relative peak area (RPA). The relative standard deviations (RSD) of the average RPA were less than 3%.

| RRT | 1 | 2 | 3 | 4 | 5 | ‾x ± SD | RSD（%） |
| --- | --- | --- | --- | --- | --- | --- | --- |
| 1 | 0.15222 | 0.15004 | 0.15592 | 0.15213 | 0.15331 | 0.15272±0.0019 | 1.23 |
| 2 | 0.60745 | 0.63181 | 0.63274 | 0.64951 | 0.63391 | 0.63108± 0.0151 | 2.39 |
| 3 | 0.37692 | 0.35421 | 0.35931 | 0.35931 | 0.37469 | 0.36504±0.0091 | 2.48 |
| 4 | 0.55745 | 0.53937 | 0.54609 | 0.54609 | 0.55574 | 0.55391±0.0120 | 2.17 |
| 5 | 0.31767 | 0.32944 | 0.33279 | 0.33279 | 0.31448 | 0.32594±0.0093 | 2.86 |
| 6 | 0.51763 | 0.51664 | 0.52873 | 0.54027 | 0.52659 | 0.52597±0.0096 | 1.83 |
| 7 | 0.96794 | 0.99059 | 0.97182 | 0.94849 | 0.97906 | 0.97158±0.0155 | 1.60 |
| 8 | 0.99697 | 1.04814 | 0.99196 | 1.00191 | 1.00278 | 1.00835±0.0227 | 2.25 |
| 9 | 0.61952 | 0.64028 | 0.62323 | 0.63425 | 0.62137 | 0.62773±0.0091 | 1.44 |
| 10 | 2.82289 | 2.76991 | 2.82366 | 2.63445 | 2.80719 | 2.77162±0.0797 | 2.88 |
| 11 | 0.43896 | 0.42688 | 0.42138 | 0.43246 | 0.41430 | 0.42679±0.0096 | 2.24 |
| 12 | 1.11569 | 1.12763 | 1.11902 | 1.11632 | 1.20081 | 1.13802±0.0040 | 2.71 |
| 13 | 1.13987 | 1.14531 | 1.12061 | 1.12031 | 1.12091 | 1.12940±0.0122 | 1.08 |
| 14 | 1.16492 | 1.15967 | 1.14337 | 1.16923 | 1.16330 | 1.16012±0.0010 | 0.85 |

**Supplementary Table 5.** Stability of the targets on relative peak area (RPA). The relative standard deviations (RSD) of the average RRT were less than 1%.

| RRT | 0h | 4h | 8h | 16h | 24h | 48h | ‾x ± SD | RSD（%） |
| --- | --- | --- | --- | --- | --- | --- | --- | --- |
| 1 | 0.16294 | 0.16923 | 0.16339 | 0.16882 | 0.16456 | 0.16538 | 0.16572±0.0027 | 0.63 |
| 2 | 0.22926 | 0.22421 | 0.22785 | 0.22849 | 0.22939 | 0.22654 | 0.22762±0.0012 | 0.87 |
| 3 | 0.43576 | 0.43221 | 0.43012 | 0.43104 | 0.43521 | 0.43221 | 0.43276±0.0023 | 0.52 |
| 4 | 0.57344 | 0.58209 | 0.57140 | 0.57109 | 0.57338 | 0.57112 | 0.57375±0.0042 | 0.74 |
| 5 | 0.65992 | 0.66825 | 0.65845 | 0.65802 | 0.65765 | 0.65487 | 0.65953±0.0046 | 0.69 |
| 6 | 1.02624 | 1.01189 | 1.02626 | 1.02622 | 1.02613 | 1.02621 | 1.02383±0.0058 | 0.57 |
| 7 | 1.05069 | 1.06292 | 1.05073 | 1.05076 | 1.05063 | 1.05063 | 1.05273±0.0050 | 0.47 |
| 8 | 1.08444 | 1.09658 | 1.08431 | 1.08436 | 1.08402 | 1.08404 | 1.08629±0.0050 | 0.46 |
| 9 | 1.16666 | 1.16666 | 1.16554 | 1.16565 | 1.16519 | 1.16517 | 1.16581±0.0007 | 0.06 |
| 10 | 1.19039 | 1.19039 | 1.18924 | 1.18929 | 1.18875 | 1.18870 | 1.18946±0.0008 | 0.06 |
| 11 | 1.23060 | 1.24267 | 1.22929 | 1.22927 | 1.22877 | 1.22868 | 1.23155±0.0055 | 0.45 |
| 12 | 1.30589 | 1.30295 | 1.30336 | 1.30227 | 1.30621 | 1.30386 | 1.30409±0.0016 | 0.12 |
| 13 | 1.33012 | 1.33659 | 1.33961 | 1.33762 | 1.33666 | 1.33832 | 1.33649±0.0030 | 0.23 |
| 14 | 1.35938 | 1.35389 | 1.35332 | 1.35051 | 1.35832 | 1.35702 | 1.35541±0.0034 | 0.25 |

**Supplementary Table 6.** Stability of the targets on relative peak area (RPA). The relative standard deviations (RSD) of the average RPA were less than 3%.

| Time | 0h | 4h | 8h | 16h | 24h | 48h | ‾x ± SD | RSD（%） |
| --- | --- | --- | --- | --- | --- | --- | --- | --- |
| 1 | 0.14936 | 0.14621 | 0.13791 | 0.14336 | 0.14702 | 0.14901 | 0.14548±0.0042 | 2.95 |
| 2 | 0.65026 | 0.65135 | 0.65162 | 0.64679 | 0.67365 | 0.68432 | 0.65967±0.0154 | 2.34 |
| 3 | 0.35421 | 0.36229 | 0.35267 | 0.34061 | 0.35693 | 0.36298 | 0.35567±0.0091 | 2.57 |
| 4 | 0.54282 | 0.52042 | 0.55613 | 0.53125 | 0.53528 | 0.55581 | 0.54029±0.0141 | 2.61 |
| 5 | 0.35755 | 0.34447 | 0.35307 | 0.33558 | 0.33456 | 0.34678 | 0.34534±0.0092 | 2.66 |
| 6 | 0.79282 | 0.80082 | 0.77584 | 0.78603 | 0.77529 | 0.83136 | 0.79369±0.0209 | 2.63 |
| 7 | 1.50004 | 1.38402 | 1.39925 | 1.39929 | 1.43272 | 1.43830 | 1.42560±0.0421 | 2.95 |
| 8 | 1.48377 | 1.46356 | 1.43352 | 1.49573 | 1.53272 | 1.54848 | 1.49296±0.0427 | 2.87 |
| 9 | 0.95157 | 0.99649 | 0.95927 | 0.97147 | 0.94876 | 1.02228 | 0.97497±0.0289 | 2.97 |
| 10 | 4.53573 | 4.63366 | 4.80772 | 4.84756 | 4.59964 | 4.82027 | 4.70743±0.1333 | 2.83 |
| 11 | 0.54734 | 0.55196 | 0.57022 | 0.53849 | 0.57353 | 0.53698 | 0.55309±0.0156 | 2.82 |
| 12 | 1.12658 | 1.14662 | 1.10209 | 1.13762 | 1.13820 | 1.13306 | 1.13069±0.0154 | 1.37 |
| 13 | 1.23064 | 1.14238 | 1.14331 | 1.14061 | 1.14536 | 1.15802 | 1.16005±0.0320 | 2.77 |
| 14 | 1.16376 | 1.24061 | 1.17308 | 1.16881 | 1.16952 | 1.18337 | 1.18319±0.0288 | 2.44 |

**Supplementary Table 7.** The concentration of rutin in ten batches in MICs of *S. oblata*. The rutin calibration curve was calculated by plotting peak areas against six different concentrations of the standard solutions (0.0126, 0.0252, 0.0504, 0.1008, 0.0152, and 0.2016 mg/mL). The calibration curve equation was *y* =2E+07*x*-11620, *R*^2^=0.9988. Different letters indicate a significant difference at *p<0.05.*

| No. | The concentration (mg/mL)（‾x ± SD） | No. | The concentration (mg/mL)（‾x ± SD） |
| --- | --- | --- | --- |
| S1 | 0.186±0.00100^b^ | S6 | 0.176±0.00153^b^ |
| S2 | 0.216±0.00322^a^ | S7 | 0.191±0.00252^b^ |
| S3 | 0.181±0.00416^b^ | S8 | 0.180±0.00208^b^ |
| S4 | 0.181±0.00306^b^ | S9 | 0.175±0.00404^b^ |
| S5 | 0.186±0.00529^b^ | S10 | 0.177±0.00265^b^ |
