# Supplementary material for: Spectrum-Effect Relationships Between the Bioactive Ingredient of Syringa oblata Lindl. Leaves and Its Role in Inhibiting the Biofilm Formation of Streptococcus suis
Source: Front Pharmacol. 2018 Jun 5;9:570. doi: 10.3389/fphar.2018.00570 (PMC5996274; doi:10.3389/fphar.2018.00570)
Supplement: Supplementary file 3 [file Data_Sheet_3.docx]

Supplementary Material 3

**Spectrum-effect Relationships between the Bioactive Ingredient of *Syringa oblata* Lindl. Leaves and its Role in Inhibiting the Biofilm Formation of *Streptococcus suis***

*Yan-Yan Liu**^1, 2+^, Xing-Ru Chen^1, 2+^, Ling-Fei Gao^1, 2^, Mo Chen^1, 2^, Wen-Qiang Cui^1, 2^, Wen-Ya Ding^1, 2^, Xue-Ying Chen^1, 2^, Bello-Onaghise God'spower^1, 2^, Yan-Hua Li^1, 2*^*

*^1College of Veterinary Medicine, Northeast Agricultural University, Harbin, Heilongjiang 150030^*

*^2Heilongjiang Key Laboratory for Animal Disease Control and Pharmaceutical Development, Harbin, China^*

*Correspondence to: Professor Yanhua Li, College of Veterinary Medicine, Northeast Agricultural University, 600 Changjiang Road, Xiangfang, Harbin, Heilongjiang 150030, P.R. China

Tel：+86 451 55191881

E‑mail: liyanhua1970@163.com (Y.-H.Li).

^†^These authors have contributed equally to this study and share first authorship.

**Supplementary Figure 1.** Alignment of the prepared CAT homology model of CAT model colored with red and three template (3CLAA, 1Q23L, 2I9DC) colored with light blue, blue, and green, respectively.


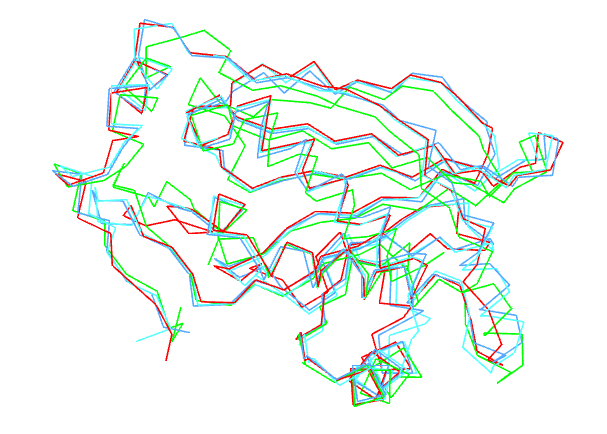


**Supplementary Figure 2.** Ramachandran Plot of CAT as analyzed by RAMPAGE.


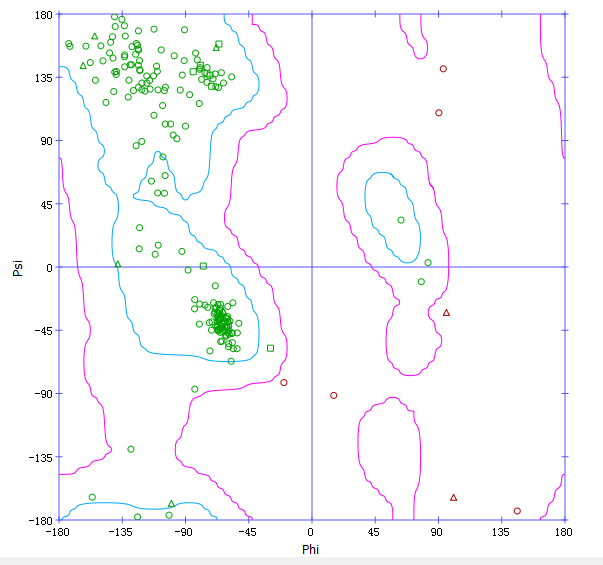


**Supplementary Figure 3.** Z-scores of the CAT model as obtained from QMEAN.

**
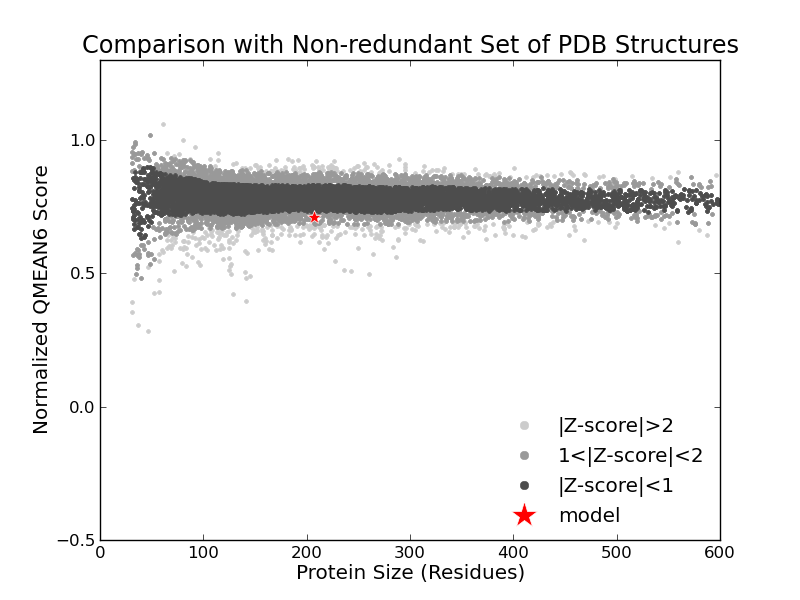
**

**Supplementary Table 1** 3D structures used as templates for homology modeling of CAT protein.

| PDB | Identity | E-value | resolution | protein name | origanism |
| --- | --- | --- | --- | --- | --- |
| 3CLA_A | 42 | 9.21E-48 | 1.75 | Chloramphenicol acetyltransferase | Escherichia coli |
| 1Q23_L | 44 | 1.65E-47 | 2.18 | Chloramphenicol acetyltransferase | Escherichia coli |
| 3I0W_A | 45 | 0.440418 | 1.73 | 8-oxoguanine-DNA-glycosylase | Clostridium acetobutylicum |
